# Engendering trustworthiness in the community: Strategies for researchers

**DOI:** 10.1177/13872877261458800

**Published:** 2026-06-26

**Authors:** Shenikqua Bouges, Esra Alagoz, Diana Gutierrez-Meza, Debra Noell, Fauzia Hollnagel, Alayna Oby, Susan Flowers-Benton, Barbara Fischer, Diane C. Gooding, Ruben L. Anthony, Fabu P. Carter, Gilda E. Ennis, Carol Van Hulle, Megan Zuelsdorff, Nickolas H. Lambrou, Taryn T. James, Emre Umucu, Cynthia M. Carlsson, Sanjay Asthana, Carey E. Gleason, Susan Racine Passmore

**Affiliations:** 1 Division of Geriatrics and Gerontology, Department of Medicine, 5232 University of Wisconsin-Madison School of Medicine and Public Health, Madison, WI, USA; 2 Wisconsin Alzheimer's Disease Research Center, University of Wisconsin-Madison School of Medicine and Public Health, Madison, WI, USA; 3 Department of Surgery, 5232 University of Wisconsin-Madison School of Medicine and Public Health, Madison, WI, USA; 4 Department of Biostatistics and Medical Informatics, 5232 University of Wisconsin-Madison School of Medicine and Public Health, Madison, WI, USA; 5 Department of Psychology, William S. Middleton Memorial Hospital, Madison, WI, USA; 6 Department of Rehabilitation, Disability Studies, and Counseling, Southern University and A&M College, Baton Rouge, LA, USA; 7 Madison VA GRECC, William S. Middleton Memorial Hospital, Madison, WI, USA; 8 Department of Neurology, 5232 University of Wisconsin-Madison School of Medicine and Public Health, Madison, WI, USA; 9 Department of Psychology, University of Wisconsin-Madison, Madison, WI, USA; 10 Urban League of Greater Madison, Madison, WI, USA; 11 Wisconsin Alzheimer's Institute, 5232 University of Wisconsin-Madison School of Medicine and Public Health, Madison WI, USA; 12 School of Nursing, University of Wisconsin-Madison, Madison, WI, USA; 13 Department of Public Health Sciences, The University of Texas at El Paso, El Paso, TX, USA; 14 South Texas VA Medical Center, Department of Health Sciences, San Antonio, TX, USA

**Keywords:** Alzheimer's disease, attitude, focus groups, trust

## Abstract

**Background:**

Despite significant advances in Alzheimer's disease treatment, underrepresentation of ethnoracialized groups in clinical trials limit the generalizability of study findings. Though mistrust in health care research is a known barrier to clinical trial participation, methods are needed to quantitate this multidimensional subjective term. This study investigated how Black participants view trustworthiness.

**Objective:**

To provide an overview of participants’ views of a trustworthy study design and investigator.

**Methods:**

This qualitative study utilized focus group discussions with Black participants 45 years and older. Transcripts were coded by three researchers by means of content analysis. After central categories were identified using concept mapping, we constructed a conceptual model of trust to reflect participants’ views of trustworthiness.

**Results:**

Participants self-identified as Black, were a mean age of 62, and predominantly female (80%). Focus group analysis revealed that a trustworthy study design and its impact as well as a trustworthy investigator were central categories of trustworthiness. Comprehensive study outlines, detailed information on the disease being studied, and sharing of study results improved participants’ willingness to be involved in studies. Participants also value researchers who are scientifically and culturally competent, knowledgeable, attentive and who engage in education and sharing comprehensive resources on diseases impacting vulnerable populations.

**Conclusions:**

These findings suggest that trustworthy features of the study design and researcher characteristics can provide a foothold to build trust with a population whose mistrust of research is well-documented. Further research on trustworthiness is necessary to develop tools to create a framework for building a trustworthy research environment.

## Introduction

Of the estimated 6.7 million Americans 65 years of age and older affected by Alzheimer's disease (AD) and AD related dementias (AD/ADRD), Black Americans have the highest prevalence.^1^ Despite the racial disparities in disease burden, Black Americans represent only 2% of participants in AD clinical trials.^1^ As we continue to develop new treatments for dementias, it is imperative to achieve more inclusive research participation to ensure generalizable findings. The adverse consequences of under-inclusion go beyond proximal health outcomes, potentially contributing to health disparities, delayed diagnosis, and unequal treatment for African Americans living with dementia.^1–3^

Limited engagement of Black participants in clinical trials lead to less awareness among Black communities of updated treatment options for AD/ADRD, as well as unclear guidance on the efficacy of newer medications and potential unknown adverse side effects.^1,2^ Moreover, under-inclusion further perpetuates mistrust among less represented populations.^3,4^ Overall, addressing the under-representation of groups at highest risk, would assure healthcare consumers, clinicians, and scientists that new therapies are safe and effective across ethnoracialized groups.^1–3^

Within the scope of this paper, the term healthcare will be used to describe medical care provided to an individual or community. Health science will encompass biomedical, medical and clinical science research which all entail the systematic investigation of health issues to improve patient, population, and public health. Though not synonymous, healthcare mistrust has been linked to distrust in clinical research among Black Americans.^4,5^ Durant et al. found higher societal distrust among Black Americans compared to White Americans, and that this distrust was associated with less trust in participants’ physician and a history of perceived discrimination in health care.^5^ Interestingly, there were no racial differences for interpersonal trust noted in Durant's study, with interpersonal trust being defined by personal experiences and interactions of individuals within healthcare or clinical research settings.^5^ Durant shared that societal distrust can be counteracted by clinical research investigators using physician referrals to build upon trust already established from the patient-provider relationship. Moreover, mistrust in health care is frequently cited as a barrier to clinical trial participation for Black Americans.^1,2,4,6^ Still there has been little attention paid to understanding how trust is created.^7^ To address this gap, we created a multifaceted recruitment approach, called the Building Bridges Program, to promote inclusive research participation as well as dementia awareness. The program was directed toward communities affected the most by this disease. In particular, we sought to clarify what researcher characteristics and features of study design engendered trustworthiness in the hope of developing paths forward for more inclusive research engagement with Black American participants.

We present here the qualitative findings of focus groups with participants in the Building Bridges Program. We hypothesized that community-informed approaches such as those emerging from community-based participatory research methods^8–11^ would be effective in building trustworthy partnerships with Black participants. Our study, therefore, applied features from a community-based participatory research framework, aiming to gain a better understanding of how Black American participants view trust in research and identify the qualities they looked for in determining a study design's and clinical scientist's trustworthiness. In this work, we follow the distinction made by other authors^12,13^ between the concepts of “trust” and “trustworthiness” and favor an approach emphasizing researchers’ responsibilities (trustworthiness) to contribute solutions to the persistent lack of diversity in participation. We focused on participant perceptions of trustworthiness and not externally measurable aspects of trustworthiness. Further, we hypothesize that mistrust in researchers is accelerated when research designs are exclusionary, and the behaviors of researchers are untrustworthy.

## Methods

### Study design and setting

The main Building Bridges program piloted interventions directed toward building trust. A smaller pilot focus group, which followed our survey recruitment study, sought to understand how Black American participants assess trustworthiness in both the study design and qualities displayed by clinical scientists.

The main Building Bridges Program was informed by Ajzen's theory of planned behavior. This larger study, sought to compare the impact of a community-based group engagement activity versus a direct one-on-one, personalized engagement activity on participant's attitudes (trust in medical researchers and/or research attitudes).^14,15^ Community engagement activities included virtual community discussions on the clinical diagnosis of dementia, symptoms to be mindful of, treatment strategies, the importance of diverse recruitment and goals of our Building Bridges Program. Our second or comparison activity provided an individualized medication review session with the study's co-investigator who is a physician. Participants were provided the opportunity to discuss their medication list, any prescriptions or over the counter drugs and learn about common medications that affect cognition. Participants had the opportunity to ask questions following both activities.

### Participants and data collection

We conducted focus groups with self-identifying Black participants aged 45 or older who were interested in sharing their views on trustworthiness and providing feedback on the Building Bridges Program. Participants who completed the interventions and surveys from the Building Bridges Program were sent an email inviting them to participate in one-hour focus groups via a virtual platform. Focus groups were conducted virtually due to the Coronavirus Pandemic which still required social distancing. Participants were divided into four discussion groups, based on their availability. Each group consisted of 2–6 participants. A race concordant moderator conducted two discussion groups, and a race discordant moderator conducted the other two discussion groups. Both moderators were part of our Building Bridges team and interacted with participants throughout the main study.

The qualitative data collection relied on focus group facilitation guides, drafted based on the analysis of our initial survey results and the literature on minoritized groups’ trust in health science and investigators. All meetings were audio recorded, transcribed, and de-identified by trained transcriptionists and imported into NVivo 12 (QSR International) for data management. This study was approved by the local Institutional Review Board and comports with the Consolidated Criteria for Reporting Qualitative Studies (COREQ).^16^

### Data analysis

We considered saturation early on in our preliminary analysis of each group as data collection was still underway and found initial categories reappeared in each discussion. In fact, there was a relatively high level of coherence across groups. As a result, we suspected saturation was reached before going into focus group 4. Our experience with the final group confirmed that saturation was reached. Therefore, our team discontinued recruitment and data collection.

Our group used team-based coding. Three of our co-authors (EA, SB, DG-M) were involved in the content analysis.^17,18^ The analysts independently reviewed transcripts, developed initial codes and met regularly to compare interpretations, discuss each category ensuring items were described and co-constructed through consensus. Our analysts resolved discrepancies and iteratively refined a shared codebook, a standard of rigor in qualitative analysis. NVIVO software was used for data analysis.

At the final stage and analysis, our team worked on a visual representation of our preliminary findings—concept mapping—further refining the map through discussion.

## Results

### Participant characteristics

Seventeen individuals contributed to our qualitative data. Participants were English language proficient, self-identified as Black/African American and aged 45 years or older (mean age = 62). More than 80% of participants self-identified as female. Table 1 provides a breakdown of the focus groups in terms of date of occurrence, facilitator, number of participants and duration of the session.

**Table 1.** Summary of focus group participants.

Focus group	Date	Facilitator	Participant totals	Duration
1	2/17/2022	SB	6	93 min
2	2/24/2022	SB	4	41 min
3	2/24/2022	DN	2	38 min
4	2/27/2022	DN	5	45 min

Focus group participants all self-identified as Black American, were an average of 62 and mainly female.

### Categories

**Figure 1.**
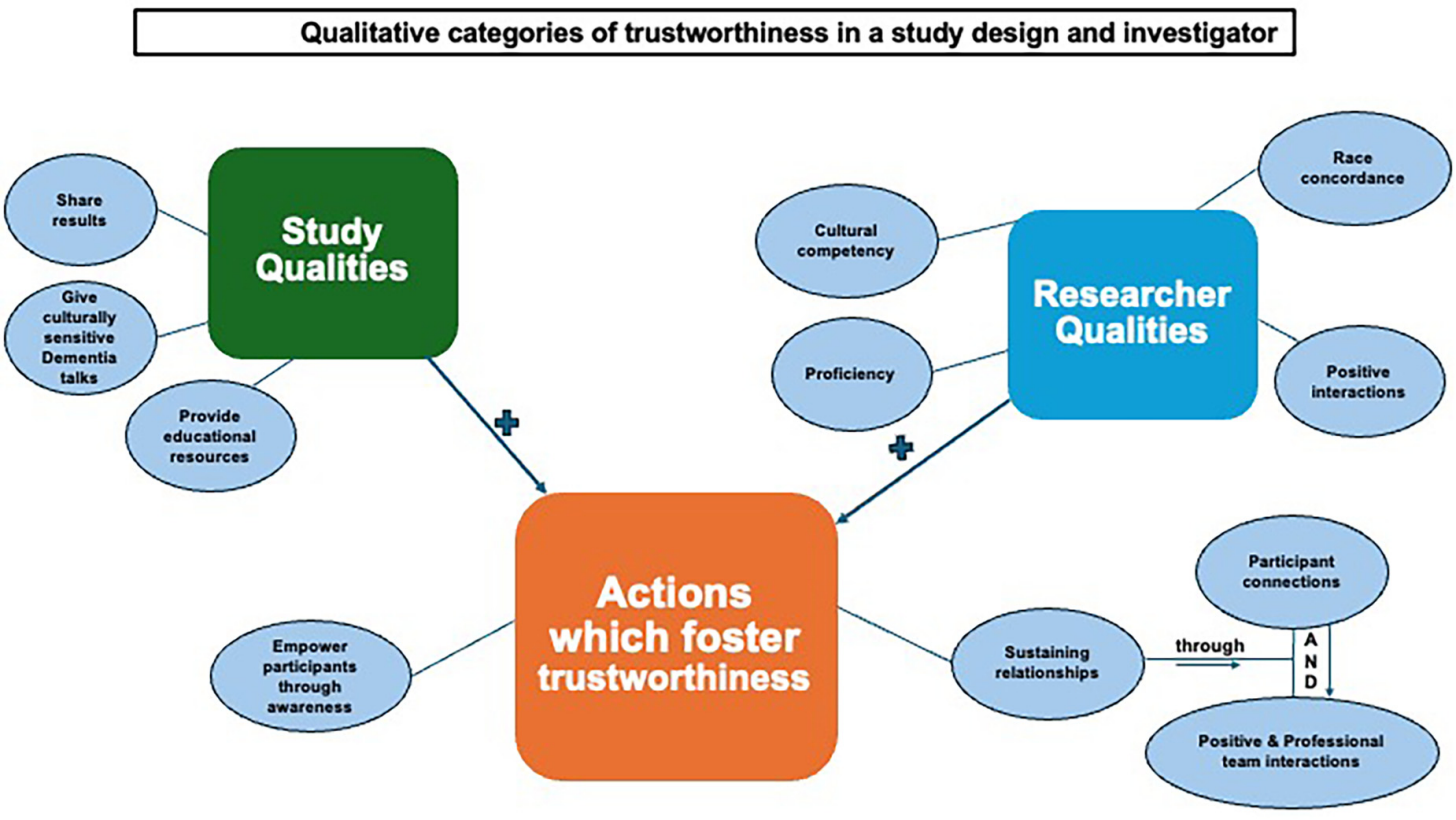
Qualitative categories of trustworthiness in a study design and investigator.

Analysis of our focus group data revealed three core categories of trustworthiness. Participants described qualities of a trustworthy study design, a trustworthy scientist, and the potential actions through which a trustworthy study design and a trustworthy investigator help to foster trust. These qualities are shown in Figure 1.

This figure displays the trustworthy qualities of a study design and the investigator(s) that were identified by the focus group participants. There were several qualities of a study design (e.g., distribution of educational resources) which served to foster trustworthiness and empower participants. Additionally, there were several qualities of an investigator (e.g., cultural competency) which contributed to fostering a sense of trustworthiness. Together, these study qualities and researcher qualities led to actions which foster trustworthiness. This, in turn, contributes to sustained relationships and partnerships through participant connections and positive and professional team interactions with community members and established organizations.

#### Category 1: Qualities of a trustworthy study design

A trustworthy study was described as a project designed to increase disease awareness in the community (through the distribution of educational resources and culturally sensitive discussions), one which clearly outlined the study goals, transparently informed participants about the research and a study which provided opportunities for engagement that shared study results back to participants. In our study, respondents were interested in receiving instructive information on the most up-to-date dementia topics and being able to access this information from a healthcare provider, scientist or a trusted community expert. The participants also valued information about research studies. Participants preferred receiving details about research studies or topic discussions in the form of advertisements, culturally sensitive information, and interesting community discussions as well as interactive question-and-answer sessions. On several occasions, respondents stressed the importance of receiving feedback on study results. Table 2 provides examples of representative quotes from participants.

**Table 2.** Participant quotes for the three core categories of trustworthiness.

Category	Factor	Representative quotes
Study qualities	Provide educational resources on dementia	“I think y’all reliable. I enjoyed the program. Y’all was very insightful. I think when I talked to you, you gave me a lot of information…. So, I really appreciate y’all…. I like the program because, like I say, I’ve probably been reading about dementia since the 90's. And I guess, like I said, I’ve seen my great auntie I guess I’m a little afraid of having dementia myself. That's why I like the information.”
Study qualities	Extensive, intriguing, and culturally sensitive dementia talks	“What would encourage me is that the topic was interesting. If I was interested in the material that was going to be presented or if it was something that I was seeking. So, I would say interesting topics would cause me to attend…. I think if it was advertised well where people were familiar with what was going to happen, I think you have to know what it's about. Maybe have a brief outline on topics or how we would handle it.”
Study qualities	Share study results with participants	“I'd like to know what the studies found. A lot of time, we participate in these research projects and then you never hear anything. What did you find? What were the conclusions? What are the practical ways this affects our community? What can we do? So, I would like to know about findings. I feel like there's so much reaching out to get information from us, but then we're not getting the findings of what you're researching. So that would be a high priority for me.”
Researcher qualities	Research study expertise	“I also look for the person to be proficient in their jobs. I worked in the hospital for 30 years…I’m looking for the person that's caring for me or giving me information to really be proficient and informative themselves. I'm more concerned with that opposed to anything else. My care, that'swhat I'm mostly concerned about.”
Researcher qualities	Knowledgeable of research study	“I think y’all reliable. I enjoyed the program. Y’all was very insightful, I think when I talked to you, you gave me a lot of information. My great auntie had Alzheimer's…And I guess for the last 10 years, maybe 20, when I was at the doctor's office, y’all always had pamphlets about dementia, and I was worried about getting dementia myself, so I’m glad I hooked up with you. So, I really appreciate y’all…I like the program because like I say, I’ve probably been reading about dementia since the ‘90 s. And I guess, like I said, I’ve seen my great auntie I guess I’m a little afraid of having dementia myself. That's why I like the information.”
Researcher qualities	Competence in understanding various racial/ethnic cultures	“If a person has, in my opinion, just because they are African American or Hispanic… they may or may not have information about me or they still could stereotype me…so I'm not going to assume that everybody has some cultural training to understand medical issues of different cultures.”
Researcher qualities	Diverse research team	“I think I’d rather have an African American myself because of that cultural and the Black experience. If you mention a certain medication or a certain procedure, the African American provider might have a family experience and y’all can relate like that…”
Researcher qualities	Create positive & memorable impressions for participants	Moderator: “So from the previous response where you say you trust more, what's changed?”“For me, the way you’ve conducted the study and the rapport that you’ve established with me and the trust that I have developed for you and sincerity and your meaning to me….that played a big part and my trust factor rising for this study, knowing that this information is being gathered with the specific purpose and it's going to be handled properly and it's going to be used for us.”
Empowerment & Relationship Building	Empower participants through disease awareness	“…the motivation for me was knowing that it was research available and to find out what I can learn to further my health. So just to have the knowledge that they exist and that it is something out there that you can learn and get familiar with it, and then you are able to attend. You got to have the awareness.”
Empowerment & Relationship Building	Form sustainable and trusting relationships	No, I just wanted to say that Ms. xxxx and Mr xxxx and I are involved in the Alzheimer's Think Cafe, trying to keep our mind motivated and sharing socially during the pandemic as well. So, we have a personal connection, and we've learned a lot about each other.
Empowerment & Relationship Building	Form sustainable and trusting relationships	“There's a level of trust…you’ve established yourself. There's credibility…and transparency.”

#### Category 2: Qualities of a trustworthy investigator

Participants listed several desirable qualities of a trustworthy investigator. Our participants valued a scientist with both study and cultural expertise, i.e., exhibiting both professionalism and cultural awareness. An investigator's trustworthiness was strengthened if participants felt an investigator was knowledgeable, thorough, and well-informed in their area of interest as well as educated on the lived experiences of individuals racialized as Black. Some respondents from our study noted increased trust and comfort with the lead investigator due to race concordance. Similarly, most participants stressed the importance of researchers displaying an array of the qualities listed below, when building rapport with ethno-racialized populations.

### Research expertise

Participants desired researchers who are well versed in their area of expertise. In our study, participants were more likely to trust investigators who were experts in their area of research and able to explain dementia to participants comprehensively without bias. They appreciated investigators who were willing to share their knowledge in community discussions, pamphlets, flyers, and presentations. For example, one of our participants noted an overall enjoyment in participating in the Building Bridges program due to the amount of information received about Alzheimer's disease and having their questions answered by a knowledgeable expert. These additional aspects of the interaction addressed the individual's fear of potentially developing dementia.

### Cultural competency

Despite participants feeling more comfortable with a race concordant staff member, it was more important for the researcher to be knowledgeable of how dementia impacts an individual identifying as Black and interact professionally throughout the course of a study. Participants discussed the nuances of cross- and intercultural interactions, noting that anyone can stereotype another person and an individual could be stereotyped by more than one characteristic. Racial identification did not equate to cultural competency in understanding how medical conditions impact diverse cultures. In addition, cultural competency, and a researcher's knowledge of communication styles with different age groups was desired when sharing the intricacies of dementia with an older population. One participant noted feelings of dismissal due to color and age from past experiences; “As an African American older woman, I’ve had physicians and nurses dismiss my concerns or not really listen to me and shove it off as well stating you’re just getting older and you have to expect those changes, rather than explaining what I can do to help with those changes or identify and accept changes.”

### Race concordance and a knowledge of participants’ “lived experiences”

Race concordance or phenotypic similarity was described by participants as representing a common set of societal interactions and an expectation of “shared historical experiences.” For example, a participant noted that the race concordant investigator was a “reflection of them.” With a similarity in race, participants expressed an expectation of corresponding medical experiences and an understanding of medical issues affecting Black Americans. Participants were aware of healthcare disparities and believed a race concordant investigator understood medical issues impacting Black Americans and was invested in helping participants better understand how common medical risk factors impacted them. Some participants noted a desire to build trust with our Building Bridges group following their positive interactions with our race-concordant team members. Participants noted a “cultural and Black experience” which provided comfort with our team broadly. Participants felt like they shared a “cultural connection” with our race concordant team members due to similar health and racial experiences which made work across team members more culturally safe. Even though a shared cultural experience with our researchers and participants was viewed as helpful in establishing fidelity with our participants, race concordance needed to be paired with cultural competency, rapport, and professionalism.

### Positive experiences through professionalism and rapport

Credibility and comfort were established with our participants through professional behavior and a co-investigator's medical background, however respondents also cited factors outside of the Building Bridges experience. Specifically, some participants referenced the long-standing foundation of building sustainable partnerships with our participants through our team's (co-author FPC) outreach work and dedication to forming long-term partnerships within our Black communities; “…*not just because of race, also because you are professional*.” Overall, research teams that valued participants with a diversity of experiences were viewed more favorably.

#### Category 3: Actions which helped foster trust while utilizing trustworthy qualities of a study design and investigator

Participants highlighted the following actions as tools to build trustworthiness and empower respondents;

1) Increasing disease awareness by distributing educational resources on conditions impacting Black Americans using lay language and clear summaries of study objectives, and 2) Working with established organizations as well as partnering with community members to;

- sustain long-term collaborations with communities most impacted by research and
- provide positive interpersonal interactions with research teams.

### Increase disease awareness

The distribution of comprehensive educational materials to study participants helped empower attendees to function as advocates for themselves, family, friends, and their communities. This form of empowerment engendered a level of comfort in participants that quelled some of their fears regarding their chances of getting dementia. The increased sense of agency and comfort contributed to rapport and trust building between research teams and the participants.

### Partnerships

As noted, trustworthiness was engendered by the provision of services, and sharing of dementia resources aimed at empowering participants to advocate for family and others. Still, participants also noted the vital role of continuing efforts to foster relationships established from prior partnerships with members of our center's outreach team. The longevity of this partnership between our participants, our larger research center and the team's demonstrated commitment to the partnership with the community were key mechanisms to establishing credibility in our research team. Participants expressed an interest in engaging in future studies due to both prior positive interactions and on-going discussions with our team, which highlight the importance of continuity.

Participants became more comfortable with and saw the research team as trustworthy as they gained a better understanding of the investigator's study goals and intent when working with a specific racialized group. This requires the investigator and the research team to be authentic and open, perhaps revealing their personal reasons for working with the Black community. Fostering a personal level of comfort and connection between the participant and research team helped to establish rapport and sustained relationships with the research community. Through these positive interactions, participants noted feeling motivated to participate in future studies and become a partner to share study information with others.

Overall, shared racial and cultural experiences helped build rapport and trust with participants, but the interpersonal interactions between our research team and respondents were noted numerous times as positive experiences which demonstrated the trustworthiness of our research team, provided comfort, and the willingness of participants to be involved in future studies. In other words, racial identity was important but not enough alone to overcome research mistrust. Positive interpersonal interactions were essential in addition to qualities of a trustworthy study design and investigator when building rapport with participants. Additionally, a programmatic and demonstrated long-term commitment to partnering with the community was valued.

## Discussion

The aim of this study was to gain an understanding of some characteristic features of trustworthiness as depicted by our focus group participants. We examined the phenomenon of trust between scientists and research participants identifying as Black or African American, and the important question of how trustworthiness is created. Our findings suggested that features of the study design, investigator characteristics, and actions promoting participant empowerment can provide a foothold to build trust with a population whose mistrust of health science research is well-documented.^3,6,19^

In alignment with the current literature, Black participants from our study expressed a willingness to participate in health science research that provides a trustworthy study design and is conducted by dependable investigators. This study found that participants described a trustworthy study design as one that provides clear, comprehensive, culturally sensitive, and informative study material presented in an engaging manner. The focus group participants also expressed a desire for a study design that featured clear study aims, transparency throughout the study, and a summary of the project results provided back to respondents. These findings are consistent with current literature highlighting study information as a facilitator to recruit Black participants into clinical studies.^6,20,21^ Also, parallel with the current literature from Portacolone and others,^6^ our participants emphasized the importance of providing culturally sensitive research material to vulnerable populations, i.e., material that outlines the significance of a study, highlights the importance of all-inclusive clinical studies and tailors the impact of a particular disease on various ethnoracialized groups.

Several researcher characteristics were highlighted to facilitate rapport. Respondents noted trustworthiness of our research team and a level of comfort established from having investigators who exemplify; a) proficiency, b) cultural competency, c) race concordance, and d) professionalism with rapport building.

### Proficiency

Participants were comfortable interacting with knowledgeable researchers in their field of study and subsequently were also willing to disseminate research information with other potential respondents. Some participants note increased comfort in asking questions once they gained a better understanding of a study. One individual, from our focus group discussion, noted a greater concern in an investigator being “proficient and well-informed” in his/her area of expertise as knowledgeableness impacts the quality of services provided to the individual.

### Cultural competency

Participants in our project highlighted the importance of having a skillful and well-informed investigator experienced in working with individuals from various backgrounds and livelihoods. In our study, participants desired information on the importance of Alzheimer's disease and how this neurodegenerative process impacted them as well as their families. These findings support previous literature highlighting the importance of clear, culturally sensitive communication with participants by investigators and aligning with some of the known barriers and facilitators to study enrollment based on current literature.^3,4,6,20,22^ For example, increasing participant awareness by providing educational discussions relevant for African American participants, taking time to clearly explain study protocols and the purpose of a study have all been cited in the literature as essential facilitators to enrolling ethnoracialized participants into clinical studies.^6,21,23–25^ In a study by Portacolone et al.,^6^ participants cited health-related information and engagement as a facilitator of trust. Participants from Portacolone et al.'s study were motivated by data suggesting that African Americans were twice as likely to develop dementia indicating the imperative of recruiting Black research participants into studies. Portacolone et al.'s respondents expressed an ardent desire to acquire new knowledge about dementia as well as other topics impacting the participants, and remained actively involved in study opportunities even after the duration of the project due to their engaging study discussions on health-related information impacting Black participants.^6^

### Race concordance

Our study design, which included a race concordant investigator, elicited notable findings. Participants expressed comfort working with race concordant staff members due to a “commonality,” “relatedness” and a stance that a similar race scientist “understand their history” and “lived experiences.” These findings correlate with current literature highlighting the importance of having a research team, whose experiences reflect those of the participants, when building rapport within various communities.^26,27^ Addison et al. found that social-emotional support increased healthy lifestyle behaviors to reduce cardiovascular disease in Black men, and support, from race concordant staff members, was one of the greatest drivers of their clinical trial participation. Emotional support from a race concordant researcher was viewed as offering a “sense of brotherhood” and “commaraderie.”^28^ In a study by Carrington Moore et al.^29^ participants felt more comfortable sharing their hardships and experiences with race concordant providers.

Participants responded to the race concordant leadership positively based on factors that led them to conclude that the researcher shared and understood their lived experiences. In addition, as stated in a study by Moore et al.,^29^ some participants felt comfortable sharing highly personal histories with race concordant staff who they believe shared similar past experiences. In Moore's study, traits of compassion, benevolence, care, and lack of judgement influenced the views of participants who described their patient care experience as a factor influencing respondents’ provider race-concordant preference.^29^

It has long been demonstrated that psychological factors such as affiliation and commonality foster positive feelings (e.g., similarity/attraction paradigm described by Byrne (1971)).^30^ Concordant cultural tiles can influence the assessment of a work colleagues’ trustworthiness within a multinational organization.^31^ Still respondents within a culturally diverse work setting, perceived organizations as also influencing their willingness to trust in various ways.^31^ Thus, trust could be built in the context of racial identity discordance when the team demonstrated components of cultural awareness and sensitivity in their fund of knowledge. Our participants noted comfort with investigators who displayed sensitivity to the participants’ cultural beliefs as well as acknowledging past historical events which created mistrust in research, health organizations, and healthcare. But again, this sense of cultural awareness also included the feature of professionalism in the research team and reputation of the unit and organization.

### Professionalism with rapport building

Respondents without strong preferences for a race concordant provider emphasized professionalism, empathy, compassion, and openness of the provider to self-educate themselves on systemic issues affecting the community regardless of race/ethnicity, as qualities valued in a provider. Our Building Bridges data uniquely highlighted the importance of professionalism combined with race concordance, cultural competency and rapport building as a powerful tool in creating a comfortable trustworthy environment for establishing trustworthiness with participants.

Interestingly, participants who engaged with our Building Bridges program through community engaging activities were more trusting of our health science team. Participants appreciated educational resources on diseases that impacted the Black American community and expressed a better understanding when information was comprehensively explained to them. Portacolone et al. also found that participants expressed a fervent desire to acquire new knowledge, noting “a better understanding to move further.” Participants in Portacolone et al.'s study, as stated previously, expressed a strong interest in being involved in discussions on various dementia topics and having the opportunity to share their perspectives.^6^ Another example… though, not directly related to trust in scientists, suggested that structural empowerment in a healthcare setting could build trust in the organization and supervisors among critical care and medical surgical nurses when nurses felt empowered. This sense of empowerment came from management involving nurses in organizational decisions and sharing information in a timely manner.^32^ The collaborations in sharing knowledge, as described in the supporting literature and our Building Bridges program, created comfort and willingness to express vulnerability for participants, which allowed them to share additional information with scientists, i.e., the researcher's openness engendered a sense of trustworthiness. This correlates with findings from Horn et al. who described increased trust by African American parents in pediatric providers who delivered a continuity of care and used a partnership building communication style or shared decision making to build rapport with families.^33^

### New insights

Among the more interesting findings, was the role of reputation and longevity in building trustworthiness. Understanding the amount of work and dedication that is required to sustain community partnerships, the data highlighted the longevity of our research group's presence as a key factor to creating trustworthiness. It provided credibility and a reputation of reliability to our research team with participants. Participants trusted our team and expressed an interest in engaging in future studies due to their long-standing positive interactions with our group.

Additionally, the data pointed to the importance of non-transactional service. To the best of our knowledge, there has been limited evidence emphasizing the importance of utilizing acts of service when establishing partnerships among diverse communities. This study adopted a service component in research when establishing rapport and trustworthiness between investigators and our communities. Our acts of services have contributed to several positive interactions and continued participation of many respondents.

Current literature highlights the importance of having a race concordant research team when recruiting diverse research participants, however, we found that cultural competency, rapport and professionalism are needed in addition to a race concordant research team, when establishing fidelity with participants.

In summary, our participants noted an appreciation of the community resources provided to them, time taken for interpersonal discussions, professionalism of our team and rapport established over time as qualities which exemplified trustworthiness.

### Limitations

Our study has several limitations, such as potential selection bias, a small sample size and limited regional representation of participant views (predominantly from Southern Wisconsin). The study may have introduced selection bias due to recruitment from an existing engagement program (Building Bridges program). Likewise, we did not have follow-up discussions with participants to further elaborate on categories. These limitations emphasize the need for additional research in larger, population-based samples with opportunities for participants to provide broader community perspectives and feedback on their responses in the future.

### Conclusions

The qualities of credible studies and scientist can be combined into three major categories. Our participants desire transparency, an allocation of educational resources, instruction, professionalism, culturally competent staff, long-standing interpersonal relationships, collaborations, and active listening from investigators. Shared similar phenotypic characteristics are important, but not the sole ingredient in establishing rapport with ethnoracialized populations. Our participants care about building sustained relationships with a health science team who take time to understand their civilization, share how the research or illness impacts various cultures, and collaboratively develop a project which will positively impact their communities. It remains essential to expand trustworthy study design efforts and for investigators to continue building sustainable, trustworthy partnerships between participants and scientists.
